# Functional materials analysis using *in situ* and *in operando* X-ray and neutron scattering

**DOI:** 10.1107/S2052252514026062

**Published:** 2015-02-03

**Authors:** Vanessa K. Peterson, Christine M. Papadakis

**Affiliations:** a Australian Nuclear Science and Technology Organisation, Locked Bag 2001, Kirrawee DC, New South Wales 2232, Australia; b Physik-Department, Fachgebiet Physik weicher Materie, Technische Universität München, James-Franck-Straße 1, 85748 Garching, Germany

**Keywords:** functional materials, X-ray scattering, neutron scattering, materials characterization

## Abstract

*In situ* and *in operando* studies are commonplace and necessary in functional materials research. This review highlights recent developments in the analysis of functional materials using state-of-the-art *in situ* and *in operando* X-ray and neutron scattering and analysis. Examples are given covering a number of important materials areas, alongside a description of the types of information that can be obtained and the experimental setups used to acquire them.

## Abstract 2

This topical review describes leading-edge functional materials research using *in situ* and *in operando* neutron and X-ray analytical techniques. Methods frequently used, experimental approaches and protocols, and results from a wide variety of materials are described, showcasing the type of information that can be obtained for these materials.

## 1. Introduction   

Functional materials are of great technological importance and central to our way of life. Such materials include those used for the storage, transport and delivery of energy, in biological and health applications, as nanostructured polymers and in construction. As environmental concerns push a broad range of advanced technologies towards sustainable and higher efficiencies, functional materials must evolve to meet these demands. Materials characterization is at the heart of functional materials research, with the relation between structure/dynamics and function paving the way for the future design of materials with improved performance characteristics.

Understanding the working mechanism at the atomic and molecular scale is the key to advancing new technologies, and central to achieving this is the study of materials during operation. As such, *in situ* and *in operando* studies are necessary in functional materials research. The *in situ* technique, often applied to materials at equilibrium, has been extended in recent years to *in operando* studies, where the materials are studied under non-equilibrium real-time conditions (or literally, in operation). New-generation neutron and X-ray sources, and associated faster instrumentation, have greatly assisted in facilitating such research.

In addition to *in operando* experiments, *in situ* experiments that map out the material response as a function of an often complex parameter space are exceptionally important to understanding material function, with parameters explored including temperature, pressure, gas pressure, pH, magnetic or electric field, and voltage or current. Such experiments may be carried out on equilibrated or non-equilibrated systems, where the latter allows the system to be examined as a function of time, enabling kinetic information to be derived. Alongside the natural division of equilibrium and non-equilibrium experimental studies of functional materials, there is a division between studies examining each material as a single component and those examining these components within a whole assembled device. Often, such experiments require specialized and complex sample environ­ments. Synchrotron and high-flux neutron instruments offer the opportunity for *in situ* non-equilibrium and *in operando* studies, with such investigations being realised by advances in both sources and instrumentation such as large, fast and sensitive detectors. Time-resolved measurements, necessary for studying kinetics, are of particular importance for the optimization of materials processing, leading to new and unique information about the molecular and atomic scale processes of functional materials.

In the present topical review, we highlight recent developments in the analysis of functional materials using *in situ* and *in operando* X-ray and neutron based analytical techniques, covering both equilibrium and non-equilibrium studies. Examples of the state-of-the-art experimental approach taken in a number of important functional materials areas are given, alongside a description of the type of information that can be obtained. These examples are not exhaustive and are drawn mostly from our own research and from work presented within the microsymposium ‘*In operando* and structure evolution – from atomic to micron’ at the 23rd Congress and General Assembly of the International Union of Crystallography in Montreal, Canada. Finally, we give an outlook for future *in operando* research.

## 2. Material-appropriate analysis tools   

*In situ* and *in operando* experiments are, by nature, demanding. In-house laboratory-based equipment (such as X-ray diffractometers) is often not shared and can therefore be customized, helping to overcome such demands. This is a real advantage over synchrotron and neutron radiation experiments at large-scale facilities, where the more difficult to access equipment is shared internationally and where instrumentation tends to be broader in its application, with most new instruments nowadays designed to be multipurpose. However, there are obvious benefits of using large-scale facilities for functional materials analysis. The higher flux offered by synchrotron sources translates directly into higher spatial and temporal resolution. Neutrons offer the advantage that the nucleus–neutron interaction allows non-destructive and bulk analysis in combination with unique isotopic information that yields differing elemental sensitivity to that provided by X-rays.

Generally speaking, it is the combination of the material under characterization alongside the temporal and spatial resolution required that dictate the analysis method and source used. The broad range of modern functional materials demand an almost equally broad number of characterization tools to understand their working mechanisms. Similar types of materials tend to be studied using similar analytical methods, and consequently the experimental techniques and *in situ* approaches that we showcase here are categorized by material type. We give a brief introduction in this section to the experimental approaches used for these materials, with the experiments themselves detailed in the remainder of the paper.

### 2.1. Polymers: nanostructural studies of films using small-angle scattering   

Polymers are ubiquitous with a broad range of properties, and these basic properties are largely determined by the microstructure formed by the polymer’s constituent monomers. Block copolymers, for example, are composed of blocks of different monomers, with their self-assembly in the solid state or in solution allowing for the formation of nano­structured materials with a variety of properties and functions, ranging from nanolithography to drug delivery. Importantly, advances in synthetic methods have enabled the rational design of block copolymers with tailored functionality and, consequently, large-scale structure characterization is of great interest for such polymeric materials. Small-angle X-ray scattering (SAXS) at grazing incidence (GISAXS) is an ideal tool to characterize nanostructured block copolymer thin films (Müller-Buschbaum, 2009 ▶). In contrast with the imaging methods (local probes) traditionally used to characterize these systems, SAXS and GISAXS yield data that are averaged over a large sample area and are thus statistically relevant. Film structures both laterally and along the normal are determined simultaneously using such methods. Varying the incident angle around the critical angle of total external reflection of the thin film allows structures within the film to be distinguished from those near the surface. Moreover, GISAXS is compatible with a number of sample environments, and high frame rates are achieved with modern solid-state detectors which allow *in operando* investigations. Similarly, small-angle neutron scattering (SANS) with deuterated polymer components enables contrast enhancement or variation, due to the pronounced difference in coherent neutron scattering length density between hydrogen and deuterium, yielding more information than SAXS. Using high-flux instruments, experiments with sub-second time resolution are possible (Grillo, 2009 ▶) and a number of dedicated sample environments have been developed, such as stopped-flow and sorption equipment.

### 2.2. Nanocrystals: structural studies using small- and wide-angle scattering   

While polymers are a good general example of the importance of nanostructure to material properties, a great many other nanostructured functional materials exist and large-scale analysis methods are equally important in studying these. Inorganic nanoparticles have large-scale structure that leads to interesting optical, electronic and vibrational characteristics, among others, with such properties dependent on the distance and interaction between the nanoparticles. Such superlattice structure can also be explored using SAXS and even wide-angle X-ray scattering (WAXS; Wang *et al.*, 2010 ▶). When the nanocrystals are in the form of thin films, GISAXS again becomes an important analytical tool (Bian *et al.*, 2011 ▶; Zhang *et al.*, 2012 ▶).

### 2.3. Porous coordination polymers: host structure studied with single-crystal and powder diffraction alongside small-angle scattering, and guest interactions studied using inelastic and quasielastic neutron scattering   

Another important class of functional materials are porous coordination polymers, also known as metal–organic frameworks (MOFs), which consist of metal ions (or clusters) coordinated by organic molecules to form structures that can be porous. It is the guest–host properties of these materials that are potentially useful for many commercially important applications, notably gas storage and separation, as well as molecular sensing. The development of analysis techniques for monitoring guest exchange in these materials is crucial for both identifying and realising future applications, with guest–host properties commonly determined using gas and vapour adsorption measurements. Coupling the structural measurements of these crystalline materials with their guest adsorption properties has revealed important structure–function relationships that enable targeted design. The host structure and how it changes with guest inclusion is of interest, with the guest–host structure of great importance to understanding function. Such measurements are best performed using traditional crystallographic tools such as single-crystal X-ray diffraction (SCXRD) and both X-ray and neutron powder diffraction (XRPD and NPD, respectively; Mulder *et al.*, 2010 ▶; McCormick *et al.*, 2014 ▶). Measurements of the guest–host structure are increasingly being performed *in situ* for a series of guests as a function of concentration within the same host sample (Peterson *et al.*, 2014 ▶).

Neutrons offer several advantages over X-rays for the analysis of guests in porous coordination polymers. Notably, the high penetration of neutrons means that complex sample environments can be used, so that the requirement for careful control of sample and gas-delivery line temperature can be more easily met than with X-rays. Samples are also more easily examined over a broader range of temperatures, expanding the parameter space for which guest–host interactions can be explored. For instance, low-temperature measurements reduce thermal motion and combine with the nuclear scattering mechanism to give highly accurate guest locations. The ability to use larger samples with neutrons means that guest–host systems can be examined with greater quantitative accuracy whereby controlled doses of a guest can be delivered, leading to concentration-dependent studies and expanding even further the parameter space explored. There is also a significant advantage in using neutrons to study guest mol­ecules containing hydrogen, particularly for mol­ecular hydrogen which is almost impossible to detect using X-rays.

Porous coordination polymer structures can extend to the nanoscale and beyond, and SAXS and SANS studies of a material’s pore structure are of particular importance to understanding function. Notably, the larger scale structure of these guest–host systems is less affected by the significant disorder that can be present as a consequence of ligand flexibility and the variety of local guest arrangements (Kauffman *et al.*, 2011 ▶). Particularly for disordered systems, perhaps the most powerful experimental method for studying guest–host interactions is spectroscopy. The dynamics of an adsorbed molecule are affected by its surroundings, and measurements of both the guest and host dynamics can be used to gain insight into the interaction between the two (Yang *et al.*, 2011 ▶). Neutrons have advantages over other spectroscopy methods here, with the nuclear scattering mechanism enabling the measurement of both structure and dynamics simultaneously. Inelastic and quasi­elastic neutron scattering (INS and QENS, respectively) provide the rotational, vibrational and diffusion dynamics of the adsorbed molecule. Additionally, vibrational density-of-state and other neutron scattering data can be used to confirm computational models, with the measured neutron scattering signal directly comparable with scattering data simulated from these computational models. The combination of computational modelling and neutron scattering is exceptionally powerful and often used to complete the picture of guest–host interactions in these materials. Neutron spectroscopy measurements are typically slower than for diffraction and therefore time-resolved measurements are relatively scarce.

### 2.4. Cement: microstructure development and hydration studied using small-angle scattering as well as inelastic and quasielastic neutron scattering   

Cement is the most widely used construction material in the world and in cement research it is the process by which the dry cement powder is transformed into the hydrated product, responsible for strength development, that is of prime importance. The development of product microstructure that gives setting cement its strength can be studied using both SAXS and SANS (Thomas *et al.*, 2009 ▶). Given the complex nature of this multiphase mixture, it is commonplace to study the hydration of a single model component rather than whole cement. Neutron spectroscopy overcomes such difficulties and provides a unique opportunity to study the hydration directly, since the incoherent neutron scattering from hydrogen dominates the data, enabling the hydrogen (even as protons) to be studied separately from the matrix as they are transferred from the water to the reaction products. Both INS and QENS are extremely useful in understanding the hydration processes through exploration of the local environment and motions of the hydrogen. The changes in the hydrogen motion observed in an INS or QENS measurement can be quantitatively correlated with the components in setting cement, including structurally bound hydrogen and constrained hydrogen in water that becomes trapped in the hardening products. To achieve temporal resolution sufficient for following the hydration process in real time, QENS data may be summed over a relatively large range of momentum transfers. Although spatial information is lost with this approach, the various hydrogen-containing products can be quantitatively identified, enabling time-resolved QENS to be used successfully to study the setting mechanism of a range of cementitious materials (Peterson, 2010 ▶).

### 2.5. Lithium-ion batteries: electrode structure and lithium distribution studied using powder diffraction and total scattering, reflectometry, neutron depth-profiling and imaging   

Electrodes are arguably the most important functional material within a battery. Both atomic and large-scale structure are important to understanding electrode function, with the main tools applied being powder diffraction and reflecto­metry. Lithium-ion batteries are the leading technology, and neutron diffraction has many advantages for research into the function of these batteries as a result of the nuclear scattering mechanism. NPD can be used to determine the crystallographic positions of the charge-carrying lithium ions, their atomic displacement parameters and their occupancies. Importantly, the high penetration of neutrons allows these to be determined from within a whole commercial-sized battery and this, coupled with the fast detection ability of modern instrumentation, enables NPD to probe in real time the bulk crystallographic changes within electrodes in functioning batteries using *in operando* studies (Sharma *et al.*, 2013 ▶; Pang *et al.*, 2014 ▶). *In operando* NPD has become increasingly important in elucidating lithium-ion insertion/extraction mechanisms, thereby leading to the understanding of lithium-ion battery function.

Nanosizing is particularly important in electrode materials, resulting in structures that are sometimes not easily studied using conventional powder diffraction. Traditional diffraction considers the long-range average structure, whereas the total scattering, as implemented in the pair-distribution function (PDF), uses both Bragg and diffuse scattering and is sensitive to local environments (Borkiewicz *et al.*, 2012 ▶). The PDF *G*(*r*) gives the probability of finding an atom at a given distance *r* from another atom and can give additional information about the local ordering and structure. To investigate the phase transformation at the local atomic scale, PDF analysis of total scattering data is exceptionally useful in studying electrode materials, where it can be used to determine local structures in a way that is sensitive to relatively long-range correlations.

Beyond the crystallographic and local scales, it is also important in lithium-ion battery research to determine the distribution of the charge-carrying lithium ions, both macroscopically and within electrode layers. Again, neutrons are important in this research, since the relatively large coherent neutron scattering from lithium is sufficient for the determination of lithium distributions within thin films and interfacial layers using neutron reflectometry (Owejan, Owejan *et al.*, 2012 ▶; Yonemura *et al.*, 2014 ▶). The relatively large neutron absorption cross-section of natural lithium enables neutron depth-profiling (Oudenhoven *et al.*, 2012 ▶) and imaging (Nanda *et al.*, 2012 ▶; Senyshyn *et al.*, 2012 ▶, 2014 ▶) to determine macroscopic lithium distributions and concentration profiles within the different components of whole batteries.

## 3. Example *in situ* and *in operando* studies   

In this section, we give example studies of functional materials in areas where *in situ* and *in operando* experiments are of particular importance. These include polymeric systems, inorganic nanocrystals, porous coordination polymers, construction materials and battery materials.

### 3.1. Polymers   

In this section, we focus on two examples, namely switchable or ‘smart’ thermo­responsive polymer hydrogels, and metal film formation during sputtering onto polymer films.

#### 3.1.1. Collapse and aggregation in thermo­responsive polymer hydrogels   

**Figure 1:**
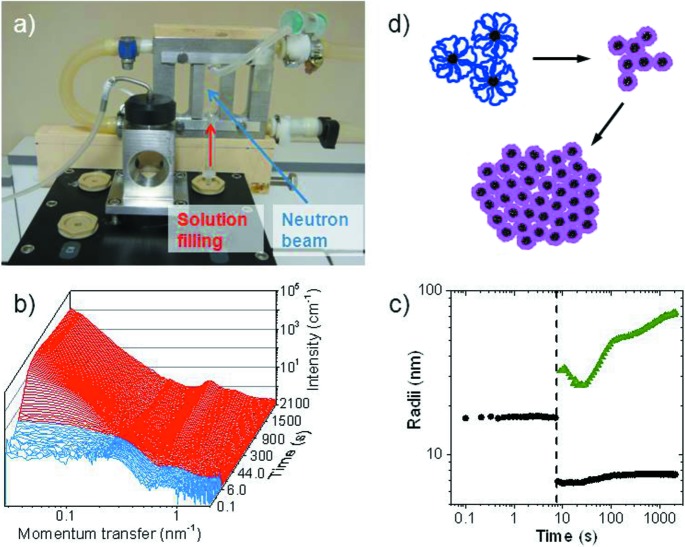
Time-resolved SANS during a temperature jump in a polymer solution. (*a*) Modified stopped-flow instrument. (*b*) SANS curves from a 200 mg ml^−1^ solution of poly­styrene-*block*-poly(*N*-isopropyl­acryl­amide)-*block*-poly­styrene triblock copolymer with fully deuterated poly­styrene blocks in D_2_O, for a temperature jump from 303 to 308 K. Temperatures below the cloud point (305.7 K) are shown in blue and above in red. (*c*) Plot showing the radii of the micelles (black circles) and the aggregate size (green triangles) with time. The dashed line marks the cloud point. (*d*) Schematic diagram of polymer collapse and aggregate formation. Adapted from Adelsberger *et al.* (2013 ▶).

Thermo­responsive polymers in aqueous solution collapse from extended coils to compact globules when the temperature is raised above their cloud point (so-called because the solution becomes turbid due to the formation of the aggregates), displaying lower critical solution temperature (LCST) behaviour, an effect that can be exploited by cross-linking the polymers. The switching time and reversibility are of great importance for applications such as controlled ultra­filtration. Time-resolved SANS provides a wealth of information on the structural changes at the cloud point. A temperature jump across the cloud point has been achieved using a modified stopped-flow instrument (Grillo, 2009 ▶), pumping the solution, held below the cloud-point, from the reservoir into a sample cell held above the cloud point. Heating by a few kelvin typically takes 100–200 s. SANS measurements can be carried out with 100 ms resolution, and the runs are typically repeated to improve statistics. A dedicated setup for such experiments is shown in Fig. 1 ▶(*a*) (Adelsberger *et al.*, 2012 ▶, 2013 ▶). SANS curves of a concentrated triblock copolymer solution, where the polymer has a thermo­responsive middle block and hydrophobic end blocks, undergo complex changes when the cloud point is crossed (Fig. 1 ▶ *b*). Detailed information is obtained on the strength and timescale of the collapse of the thermo­responsive block which makes up the micellar shell and on aggregate growth (Fig. 1 ▶ *c*). Such investigations have been carried out on various polymeric architectures (Meier-Koll *et al.*, 2012 ▶; Adelsberger *et al.*, 2012 ▶, 2013 ▶; Jaksch *et al.*, 2014 ▶).

#### 3.1.2. Metal film preparation on nanostructured polymer surfaces   

Nanostructured block copolymer thin films have been proposed as templates, guiding metal nanoparticles onto solid surfaces to create nanopatterns. Gold, for instance, has unique optoelectronic properties, and gold cluster assemblies are therefore promising candidates for solar cells and reflective or antireflective coatings. Knowledge of the mechanisms involved in nanoparticle formation and growth during metal sputtering onto nanostructured block copolymer thin films is important for identifying appropriate metal sputtering conditions. Early experiments were carried out in a stop–sputter mode, in which the metal was sputter-deposited onto the substrate at a certain deposition rate for a few seconds and two-dimensional GISAXS maps were collected after each sputtering step (Metwalli *et al.*, 2008 ▶; Kaune *et al.*, 2009 ▶; Buffet *et al.*, 2011 ▶). Recently, real-time GISAXS data were acquired *during* the sputtering process (Schwartzkopf *et al.*, 2013 ▶; Metwalli *et al.*, 2013 ▶). In both types of experiment, and in combination with *ex situ* atomic force microscopy, structural information is gained regarding the shape, size and distribution of the metal nanoparticles, as well as their spatial ordering on the surface and the metal distribution along the film normal.

The *in situ* growth of cobalt metal on a laterally nano­structured polystyrene-*block*-polyisoprene diblock copolymer thin film was investigated in a real-time sputtering experiment (Metwalli *et al.*, 2013 ▶). GISAXS images were taken during the sputtering process with a time resolution of 1 s at a sputter rate of 0.8 nm min^−1^. The GISAXS data identified the onset of metal film formation through intensity oscillations along the film normal. The film thickness could be deduced and compared with the nominal sputter rate. Out-of-plane reflection peaks observed in the pure block copolymer thin film were reproduced in data from the cobalt nanoparticles, allowing three regimes to be distinguished: nucleation of atoms resulting in nanoparticle formation preferentially in the polystyrene domains, particle growth, and coalescence of neighbouring metal aggregates into a quasi-uniform metal layer. Other *in situ* real-time GISAXS experiments have studied metal deposition onto amorphous silicon oxide (Schwartzkopf *et al.*, 2013 ▶) and materials for organic light-emitting diodes (Yu *et al.*, 2013 ▶).

### 3.2. Inorganic nanocrystals   

The functional properties of inorganic nanoparticles are dependent on the type of superlattice formed as a result of the interaction between the nanoparticles. These parameters may be changed by pressure, ligand, or solvent vapour, which influence the arrangement of the nanoparticles. Under certain conditions, collective optical and electronic properties may be obtained and controlled.

#### 3.2.1. Structural changes as a function of pressure   

*In situ* SAXS and WAXS measurements using a high-pressure cell, *e.g.* a diamond anvil cell (DAC), allow the relationship between nanoparticle distance and structure to be explored. To achieve these measurements, a number of experimental parameters need to be modified, such as the X-ray energy to minimize absorption by the diamond windows. In wurtzite CdSe supercrystals, the interparticle spacing was measured up to 10 GPa and its variation related to changes in the atomic structure of the nanoparticles (Wang *et al.*, 2010 ▶).

#### 3.2.2. GISAXS vapour sorption/desorption studies   

**Figure 2:**
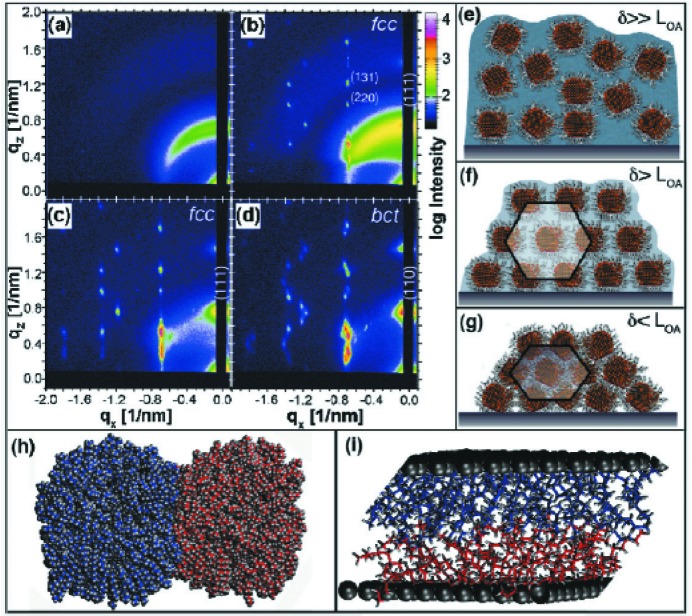
*In situ* GISAXS patterns and corresponding structures of solvent vapour-mediated transformations in a PbS nanocrystal superlattice. (*a*) and (*e*) Disordered film in a saturated octane vapour environment. (*b*) Initial superlattice nucleation in a subsaturated vapour environment. (*c*) and (*f*) Face-centred cubic (f.c.c.) nanocrystal superlattice formed by drying the film. (*d*) and (*g*) Body-centred tetragonal (b.c.t.) nanocrystal superlattice formed by drying in the presence of He purge. (*h*) Molecular dynamics snapshot of two nanocrystals and (*i*) of ligands between facets of proximate nanocrystals. δ and *L*_OA_ denote the interparticle distance and length of the oleic acid ligand, respectively. Reprinted with permission from Bian *et al.* (2011 ▶). Copyright (2011) American Chemical Society.

Solvent can change the interaction between nanocrystals and represents another means for manipulating properties (Fig. 2 ▶). Films of PbS or PbSe nanocrystals modified with oleic acid form a body-centred tetragonal (b.c.t.) structure. When exposed to octane vapour, this undergoes a (reversible) symmetry transformation to a face-centred cubic (f.c.c.) structure within a few minutes, as shown using *in situ* GISAXS (Bian *et al.*, 2011 ▶). In another *in situ* GISAXS experiment, the symmetry transformations on removing solvent from a swollen, and therefore disordered, film were addressed. The choice of solvent mediates the ligand–ligand interaction and modulates the timescale of interdigitation, and the solvent evaporation rate plays an important role in the final structure. Time-resolved GISAXS measurements are thus ideal to investigate the structural changes, with the results paving the way for engineering quantum crystals with desired properties.

The self-assembly of non-spherical nanocrystals was investigated using Pt_3_Cu_2_ nanoctahedra capped with oleylamine (Zhang *et al.*, 2012 ▶). *In situ* GISAXS revealed crystallization during controlled solvent evaporation in real time. As the suspension in hexane dried slowly in a solvent vapour treatment (SVT) chamber, the broad intensity distribution in the GISAXS data transformed into discrete Bragg spots which continuously changed position during drying. A critical concentration of nanocrystals was determined at which crystallization sets in, corresponding to a Kirkwood–Alder transition. The body-centred cubic (b.c.c.) lattice formed at the disorder-to-order transition shrinks down to the limit of steric hindrance, where the nanoctahedra arrange tip-to-tip. Subsequent swelling in solvent vapour reveals the process to be reversible and so defects present after initial drop-casting may be annealed. Thus, ‘smart’ environmentally adaptable mat­erials for application in sensors and catalysis may be developed.

### 3.3. Porous coordination polymers   

Porous coordination polymers, also known as metal–organic frameworks (MOFs), are a large class of often porous mat­erials with unprecedented chemical and structural diversity as a consequence of their relative ease of synthetic modification, allowing targeted modulation of pore shape and size, as well as chemical functionality. Consequently, MOFs have become popular as alternative materials for applications including gas and liquid separation, energy storage, nanotechnology and biological applications. In particular, MOFs acting as porous sorbents are potentially of importance in energy systems, with these materials able to separate and reversibly store energy-relevant gases, including H_2_, CH_4_ and CO_2_, from a range of industrially relevant mixtures.

The majority of diffraction experiments exploring guest–host interactions in porous coordination polymers are *in situ* in nature, although the approach used varies in accordance with experimental need. Most commonly, the materials are first analysed with their pores empty (activated state), before the sample is remeasured at equilibrium after the introduction of guest molecules.

#### 3.3.1. SCXRD vapour sorption studies   

Peterson *et al.* (2014 ▶) investigated *in situ* the structure of a range of guest molecules within the same single crystal of Cu_3_(1,3,5-benzene­tri­carboxylate)_2_, a well known porous coordination framework with a broad range of applications. SCXRD data were collected on a crystal positioned inside a polyimide capillary with the top open. The capillary was mounted in the centre of a cryostream and the bottom either closed or connected to a tube of dry helium or helium/vapour flow. Data were collected at 293 K for the material equilibrated with different guest vapours, with the vapours introduced by bubbling helium through a glass vial of the selected guest liquid. The crystal was desolvated at 473 K under dry helium flow between the different vapours. Full data sets were collected for cyclohexane, methanol, ethanol, propan-1-ol, propan-2-ol, toluene, acetonitrile and tetrahydrofuran. Structural solution and refinement were successful for all vapour–host data and revealed the host structure, with difference Fourier methods used to locate the guests within the three pores, including interaction with the coordinatively un­saturated Cu^II^ sites. The structural measurements correlated with the vapour sorption measurements, delivering a detailed understanding of the underlying mechanisms responsible for adsorption behaviour and revealing a strong thermodynamic influence (both enthalpic and entropic), as well as size-exclusion effects.

#### 3.3.2. NPD gas adsorption studies   

Using NPD, McCormick *et al.* (2014 ▶) examined the novel ultramicroporous material Cu_3_[C(CN)_2_(CONH_2_)^−^]_4_. The 0.35 nm nanotube-like channels of this material and the unprecedented concentration of coordinatively unsaturated Cu^II^ sites result in selectivity of CO_2_ over CH_4_, which is useful for the industrial separation of CO_2_ from natural gas. As neutron diffraction intensity does not reduce with scattering angle, relatively more fine structural information is gained than using X-rays and this, when combined with the neutron’s isotopically dependent information, provides details of the CO_2_ orientation in this mat­erial. Furthermore, neutrons readily penetrate the complex sample environment required for controlling the temperature of the host at the same time as gas delivery, making it easy to cover a range of temperature from very cold (about 10 K) to more moderate working conditions (313–348 K). The neutron penetration also provides information about the more industrially relevant ‘bulk’ properties of the material and aids in accurate dosing with a known number of guest molecules.

**Figure 3:**
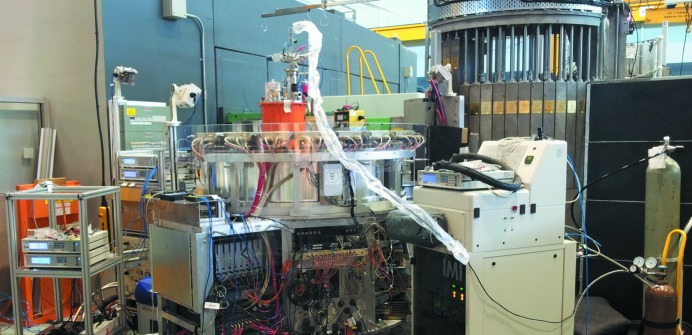
NPD experimental setup for examining porous sorbents, shown for the ultramicroporous Cu_3_[C(CN)_2_(CONH_2_)^−^]_4_ experiment of McCormick *et al.* (2014 ▶). The setup features full remote control once the sample is in place, with temperature control of the sample and gas dosing line.

NPD data were collected for ∼1 g of desolvated material, which was sequentially dosed with two concentrations of CO_2_. While this approach has been used previously, this was the first time gas dosing, evacuation and measurement were achieved remotely from the instrument cabin. The sample cell was connected to a gas-delivery stick custom-designed for use with a top-loading cryofurnace. The sample was isolated with a valve that was opened upon connection to the gas-delivery stick, which has an isolation valve located external to the cryofurnace. A customized and automated manometric gas-delivery system was attached to the gas-delivery stick, enabling gas delivery to or evacuation of the sample. The temperature of the sample and gas-delivery line was controlled independently of the cryofurnace, allowing the sample to be left in place throughout the experiment (Fig. 3 ▶). Data for the empty and carbon-dioxide-loaded sample were collected for 30 min at 15 K, with the sample warmed to 300 K prior to dosing and the delivery line kept above the boiling point of CO_2_ at that pressure. After gas adsorption, identified by a zero pressure reading, the sample was cooled over approximately 1 h, ensuring diffusion of the CO_2_ molecules to their thermodynamic equilibrium positions and minimizing disorder. This experimental setup allows remote switching between types of gases and sample regeneration.

Data were analysed using a combination of Rietveld and difference Fourier methods and molecular dynamics simulations. A close packing of single rows of CO_2_ in the tubular nanostructure of Cu_3_[C(CN)_2_(CONH_2_)^−^]_4_ was revealed, in which the CO_2_ was shown to interact with the Cu^II^ sites, revealing the mechanism of selectivity for CO_2_.

**Figure 4:**
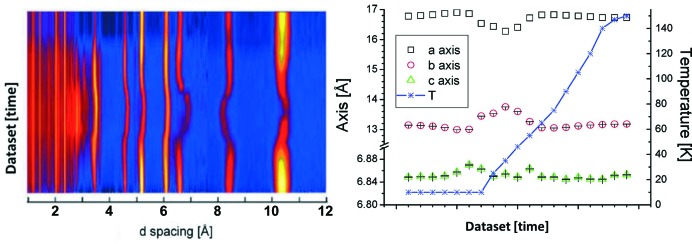
Non-equilibrium NPD data of molecular deuterium in Cr(OH)(1,4-benzenedicarboxylate). (Left) NPD data as a function of time, collected during loading at 25 K, cooling to 10 K and subsequent heating to 150 K, under a constant pressure of 2.0 bar of molecular deuterium. (Right) Lattice parameters extracted from these data. Reprinted and adapted with permission from Mulder *et al.* (2010 ▶). Copyright (2010) American Chemical Society.

Advances in neutron instrumentation, particularly large and fast area detectors, have led to the opportunity to collect real-time data. While *in situ* NPD data during guest adsorption by porous materials have been collected on a timescale of minutes using the setup in Fig. 3 ▶, the first non-equilibrium measurements were reported by Mulder *et al.* (2010 ▶). In this work, time-of-flight NPD was used to elucidate the storage mechanism of molecular hydrogen in the porous Cr(OH)(1,4-benzene­dicarboxylate) material. *In situ* NPD data were recorded every 10–15 min during loading and unloading of molecular deuterium at various temperatures (Fig. 4 ▶).

This work also examined the Cr(OH)(1,4-benzene­dicarboxylate) structure equilibrated with known amounts of deuterium gas using a less automated approach than that used by McCormick *et al.* (2014 ▶). The delivery of gases with a low boiling point such as deuterium is less challenging experimentally than for gases with a higher boiling point (such as CO_2_), because of the reduced heating of the system required to avoid freezing of the gas in the delivery line. Data for the non-equilibrium system collected during gas loading were obtained by exposing the sample to a constant pressure of 2.0 bar (1 bar = 100 000 Pa) of molecular deuterium. Here, neutrons are needed to measure the structure of hydrogen in the system, where insufficient scattering power is provided by X-rays which provide information dominated by the electron-heavy host. Rietveld refinements were performed using difference Fourier methods, with the deuterium molecule modelled as a spherically averaged point scatterer. This work found expected and significant ‘breathing’ of the structure upon hydrogen loading and release. Interestingly, the breathing was found to be in the direction of the larger pore openings and opposite to that observed for water uptake. Such guest-dependent behaviour is best captured by studying the system under non-equilibrium conditions, where the system is performing close to its working conditions as a hydrogen-storage material.

#### 3.3.3. SANS gas adsorption studies   

The flexible pillared layered cyanide material Ni[1,2-bis(4-pyridyl)ethylene][Ni(CN)4] (NiBpeneNiCN) selectively adsorbs CO_2_ over N_2_. Interestingly, the rapid rise in the uptake of CO_2_ by this material during separation from N_2_ is accompanied by a dynamic structural transition, and this was investigated using *in situ* neutron diffraction in combination with IR spectroscopy (Kauffman *et al.*, 2011 ▶). Here, the crystallographic detail of the material could not be obtained using traditional powder diffraction, due to peak broadening, presumably as a result of disorder. A SANS instrument was used to measure the 002 lattice spacing, which changed from ∼12.46 to 13.28 Å upon saturation of the material with CO_2_ but not with pure N_2_. This work revealed that the lattice changed only upon exposure to the preferentially adsorbed CO_2_ and not N_2_, indicating that the structural response observed in the 50:50 gas mixture is driven by CO_2_.

#### 3.3.4. QENS gas adsorption studies   

The dynamic information obtained through neutron scattering is isotopically dependent, and neutron spectroscopy allows direct measurement of the local environment and the diffusional transport of the guest within the host. Using neutrons, both structure and dynamics can be measured simultaneously, enabling insights into the geometry of the guest motion and gaining details of the diffusion mechanism. Neutron spectroscopy measurements are typically slower than for diffraction, and therefore time-resolved measurements are relatively scarce. *In situ* neutron spectroscopy experiments investigating porous materials have been carried out for systems equilibrated at a variety of temperatures and guest concentration or pressure. Yang *et al.* (2011 ▶) used QENS to investigate concentration-dependent CO_2_ and CH_4_ diffusion in the highly stable porous material Zr_6_O_4_(OH)_4_(1,4-benzenedicarboxylate)_12_, known for its CO_2_ selectivity. An activated sample was loaded into a rectangular aluminium cell fitted with a gas inlet allowing *in situ* adsorption, with the gas amounts determined mano­metrically, in an approach similar to that used by others such as Mulder *et al.* (2010 ▶) and with a more basic approach than that of McCormick *et al.* (2014 ▶). All parts of the gas-delivery system and sample were above 230 K during adsorption, with QENS data for the material with and without CO_2_ collected without further cooling (*i.e.* at 230 K). The sample was re­activated *in situ* and data then collected as a function of CH_4_ concentration. In contrast with CH_4_, CO_2_ is a totally coherent neutron scatterer, and the diffusion constant obtained using QENS for this molecule was derived from the reduction in elastic neutron scattering as a result of diffusion. Hence, this work derived the experimental self-diffusivity of CH_4_ and the transport diffusivity of CO_2_ in the material. The QENS results were calculated using molecular dynamics simulations, and in this way the mechanism of diffusion for both gases within the material could be extracted and was found to be significantly concentration dependent. The coupling of neutron scattering results with computational calculations is a powerful combination, often yielding the exact details of local processes, with the computational model validated against the experimental result for the average system.

### 3.4. Cement   

Globally, our use of cement in construction is second only to that of water. Understanding the physico­chemical processes by which the dry powder reacts with water to yield the strong final material allows the optimization of physical attributes such as strength and durability. Cement is becoming more complex as higher performance and more environmentally friendly materials are developed. Neutrons are useful for studying cement, where their penetration allows detail from the bulk to be gained and the incoherent scattering from hydrogen can be used to study the hydration process directly, despite the complexity of the matrix. Time-resolved neutron spectroscopy and SANS have been used to study the fundamental hydration processes and mesoscopic structural changes. The insights gained from relating such neutron studies to laboratory-based measurements of heat evolution have pioneered a new approach to interpreting laboratory-based calorimetry data of cementitious systems and allowed the limitations of such measurements to be understood.

#### 3.4.1. Time-resolved neutron spectroscopy   

**Figure 5:**
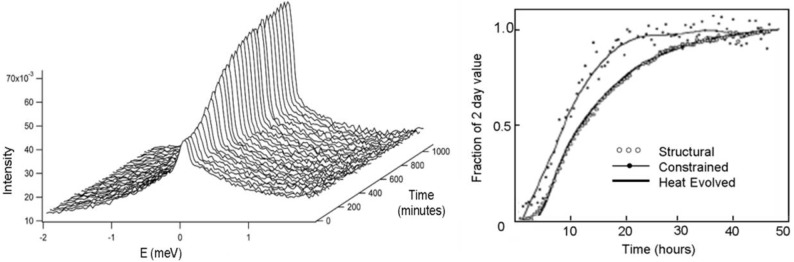
Time-resolved data for the hydrating main component of ordinary Portland cement (tricalcium silicate). (Left) QENS spectra. (Right) Time evolution of the constrained hydrogen and immobile hydrogen (structural) components obtained using QENS (left), alongside the total heat evolved as measured using calorimetry. Adapted from Peterson (2010 ▶).

Tricalcium silicate is the main component of ordinary Portland cement, and it hydrates to form crystalline calcium hydroxide and ‘calcium silicate hydrate’, the latter product having variable stoichiometry. A typical time-resolved QENS data set for hydrating tricalcium silicate is shown in Fig. 5 ▶, where the central peak at zero energy transfer arises from immobile hydrogen in the reaction products, *i.e.* calcium hydroxide and some types of calcium silicate hydrate, and increases over time. In particular, calcium silicate hydrate is of great importance as it is the component responsible for strength development. In this experiment, the main component of cement, tricalcium silicate, was mixed with water and spectra collected every 33 min, with the data collection beginning 30 min after initial mixing.

The hydrogen component that is considered immobile on the timescale accessible by the spectrometer, known as ‘structural’ hydrogen, closely tracks the evolution of heat, as measured by calorimetry, for the first 2 d (Fig. 5 ▶). Two different calcium silicate hydrates have been found in setting cement, and both can be measured using QENS. Of the reaction products, the ‘structural’ QENS component is associated with the formation of all of the calcium hydroxide and one of the calcium silicate hydrates. The less mobile hydrogen component, ‘constrained’ hydrogen, is found in interlayer water in the smallest pores and adsorbed onto surfaces. Notably, this QENS component is associated with the formation of a high-surface-area calcium silicate hydrate phase. Hence, QENS measures all hydration products and not just those associated with the evolution of heat. Kinetic information on the formation rate of various products can be derived from such measurements and is invaluable for understanding the development of important properties such as strength (Peterson, 2010 ▶).

Although QENS is the major time-resolved neutron spectro­scopic technique used to study cement hydration, INS has also been used for this purpose. FitzGerald *et al.* (1999 ▶) first used INS to measure quantitatively the formation of calcium hydroxide during the hydration of a cementitious material by comparison of the INS spectrum for the hydrating sample with that for a calcium hydroxide reference material. The work enabled kinetic parameters for the hydration to be derived. Importantly, combining the INS results with the QENS results allowed the hydrogen content of the calcium silicate hydrate to be established. Extension of the time-resolved work to include the temperature dependency of calcium hydroxide evolution in the non-equilibrium system revealed that the hydrogen content of the calcium silicate hydrate decreased significantly at increased curing temperature, likely contributing to the altered strength.

#### 3.4.2. Time-resolved SANS   

**Figure 6:**
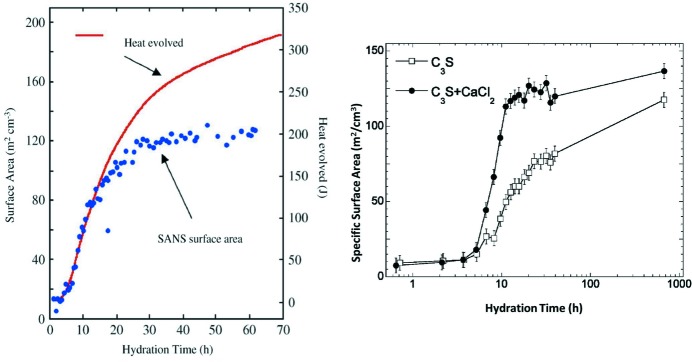
Surface-area development of hydrating tricalcium silicate. (Left) Surface-area development, shown alongside heat output as measured using calorimetry. Reprinted with permission from Allen & Thomas (2007 ▶). Copyright (2007) Elsevier. (Right) Surface-area development with (circles) and without (squares) calcium chloride. Reprinted with permission from Thomas *et al.* (2009 ▶). Copyright (2009) American Chemical Society.

The surface area of cement increases during setting, and real-time SANS and SAXS have been used to follow the fractal microstructural development in hydrating cement. Although the microstructural development measured by SAXS and SANS has been shown to follow the heat output initially, as with QENS this does not continue over extended periods (Allen & Thomas, 2007 ▶). The dense (inner product) calcium silicate hydrate phase, produced after approximately 24 h, has a morphology that is invisible to SANS and SAXS. These techniques are therefore easily able to follow the development of the less dense (outer product) calcium silicate hydrate, important in the development of strength. Such experiments have been used to examine, amongst others, the effect of additives such as the accelerant calcium chloride on the microstructural development, as shown by Thomas *et al.* (2009 ▶; Fig. 6 ▶).

### 3.5. Battery materials   

The general aim of battery research is to improve energy and power density, manufacturing costs, safety and cycle life, but there is a need to meet these challenges in an environ­mentally sustainable way. A common starting point is to examine the cathode function which, in the leading technology, the lithium-ion battery, can account for as much as 40% of the battery cost. Following the commercialization of the first lithium-ion battery in 1991, the *in situ* structural analysis of battery materials has increased rapidly, mostly through the use of powder diffraction. In particular, the early research on whole batteries equilibrated at a particular state of charge has moved on to the rapid acquisition of data during the charge and discharge of the battery. Such *in operando* studies have led to deep insights into the mechanism of charge transfer within batteries, paving the way for new materials to be developed, but also pointing to ways of best operating a battery, such as by defining charge and current limits.

#### 3.5.1. *In operando* synchrotron XRPD and pair-distribution function analysis   

**Figure 7:**
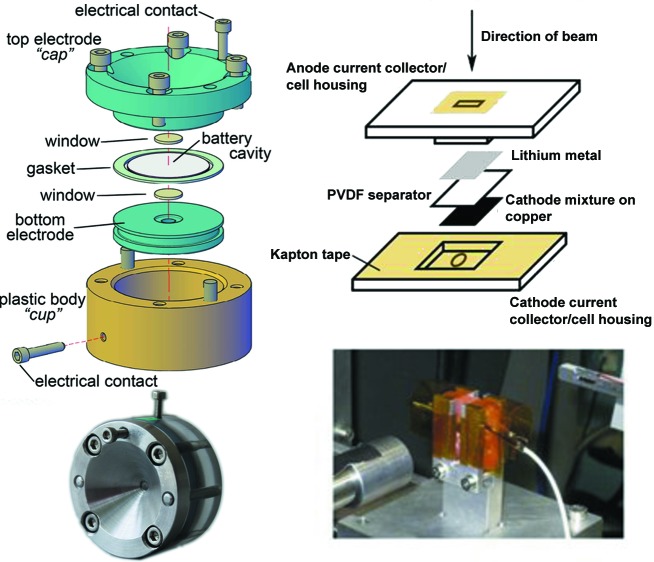
Specialist electrochemical cells for use in *in situ* transmission synchrotron XRPD experiments. (Left) The AMPIX cell for use in PDF experiments (Borkiewicz *et al.*, 2012 ▶). (Right) Schematic diagram of a cell for traditional synchrotron XRPD [Brant *et al.* (2013 ▶). Reprinted with permission. Copyright Elsevier (2013)]. Exploded views (top) and assembled cell photos (bottom) are shown.

As structural changes in both the anode and cathode play an important role in overall battery performance, *in situ* X-ray diffraction has become an essential tool in rechargeable lithium-ion battery research. Given the importance of this research, particularly in understanding the phase and lattice-parameter evolution of electrode materials during charge and discharge, it was necessary to develop specialized electrochemical cells for experiments. Brant *et al.* (2013 ▶) presented a simple *in situ* cell design for this purpose (Fig. 7 ▶, right-hand side), derived from various *in situ* electrochemical cells developed over the last three decades. Importantly, the design uses components that are routinely available and can be machined in-house.

**Figure 8:**
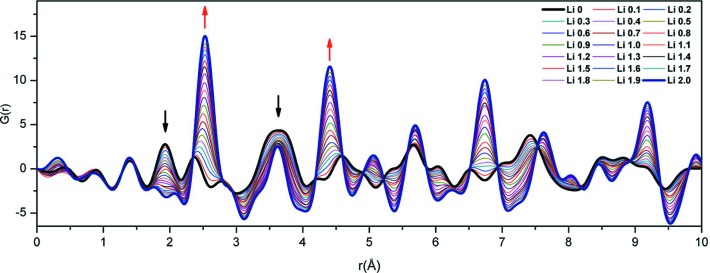
*In operando* PDFs for the CuF_2_ cathode during the first discharge, with initial and end states highlighted in bold. Black and red arrows indicate a decrease and an increase in intensity, respectively. Reprinted with permission from Hua *et al.* (2014 ▶). Copyright (2014) American Chemical Society.

XRPD pair-distribution function (PDF) analyses of electrodes have been performed *in operando* during battery cycling (Hua *et al.*, 2014 ▶). This study examined the CuF_2_ cathode, of particle size ∼9 nm, where cathode pellets were assembled into the specialist AMPIX cell (Borkiewicz *et al.*, 2012 ▶), shown in the left-hand part of Fig. 7 ▶, and Li metal was used as the counter-electrode. Total scattering data were collected in transmission geometry. The collection of high-quality total scattering data for PDF analysis requires careful subtraction of data for components other than the material of interest. In this study, data for a reference cell containing all cell components in the same mass ratio except the active material were collected under the same experimental conditions. In addition to background subtraction, further corrections for sample self-absorption, multiple scattering, X-ray polarization and Compton scattering were included to obtain the normalized total scattering structure function, and the PDF was generated *via* its direct Fourier transformation (Fig. 8 ▶). The data give a detailed insight into the local structural changes occurring in the cathode during electro­chemical function. Data at the beginning of the discharge were dominated by CuF_2_, with peaks at ∼1.9 and 2.3 Å corresponding to equatorial and axial Cu—F bonds, respectively, reflecting a Jahn−Teller distortion for Cu^2+^. Broad features at ∼3.6 Å correspond to distances between Cu^2+^ cations, and between Cu^2+^ and F^−^ ions, in neighbouring octahedra. The PDF at the end of the discharge has peaks at ∼2.6, 3.6 and 4.4 Å, corresponding to Cu—Cu distances in the first, second and third coordination shell, respectively, for face-centred cubic (f.c.c.) Cu metal. The transformation from CuF_2_ to Cu is also evident in intensity changes.

#### 3.5.2. *In operando* NPD   

**Figure 9:**
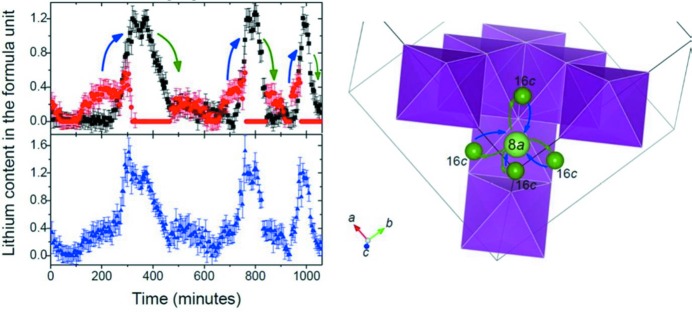
*In operando* NPD study of the Li_1+*y*_Mn_2_O_4_ cathode. (Left) Time-dependent occupancy and location of lithium at the 8*a* and 16*c* crystallographic sites (black and red, respectively; total shown in blue). (Right) The diffusion path between the crystallographic sites during charge (green arrows) and discharge (blue arrows). Adapted from Sharma *et al.* (2013 ▶).

The possibility of using *in operando* powder diffraction to study not only the reaction mechanism and lattice-parameter evolution of electrodes, but also the insertion/extraction mechanism of charge carriers within these, is highly useful in battery development. In particular, NPD can be used to gain insights into lithium within electrodes, and consequently *in operando* NPD is attracting increasing attention in the lithium-ion battery research community. Such information is difficult to extract, and until recently was only demonstrated under equilibrium conditions with long collection times. The first real-time NPD *in operando* experiment was performed by Sharma *et al.* (2010 ▶) on a commercial lithium-ion battery. This work established the phase evolution of the cathode and anode. Since then, developments in experimental and analytical approaches have enabled the lithium occupancy and location within the cathode and anode to be extracted using *in operando* NPD, as demonstrated first by Sharma *et al.* (2013 ▶). This work used a prismatic battery with dimensions 5 × 36 × 70 mm composed of an aluminium casing, aluminium and copper current collectors, a graphite anode and the cathode mixture. Data were collected every 5 min for 21 h. Full Rietveld analysis of the data revealed the lithium location and amount during charge/discharge (Fig. 9 ▶). This work provided an unparalleled insight into the function of the cathode, explaining its relative ease of discharge compared with charge. The experiment paved the way for further work by Pang *et al.* (2014 ▶) using custom-designed batteries with an even further improved signal, achieved in part due to the use of deuterated electrolytes.

#### 3.5.3. *In operando* neutron reflectometry   

**Figure 10:**
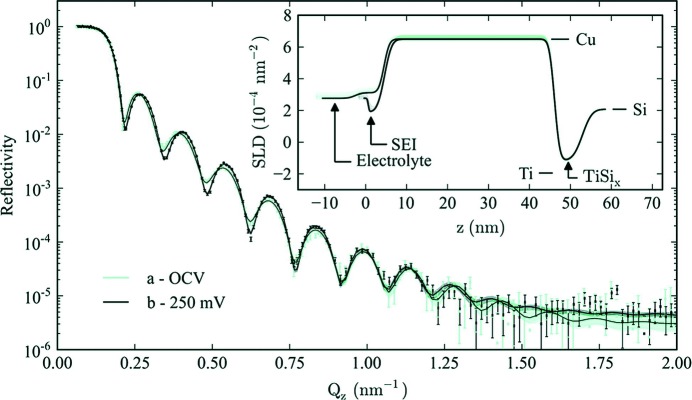
Neutron reflectivity data for a cell at open-cell voltage (OCV) and after ten cycles. Solid lines are the best fit. (Inset) The scattering length density (SLD) of Si, Cu, Ti and electrolyte, with SEI and TiSi_*x*_ layers. Reprinted with permission from Owejan, Owejan *et al.* (2012 ▶). Copyright (2012) American Chemical Society.

Integral to the function of a lithium-ion battery is the formation of the solid–electrolyte interface (SEI) layer, a self-forming passivation layer generated as a result of electrolyte instability with respect to the anode chemical potential. The SEI layer generates sufficient electronic resistance to limit further electrolyte decomposition. However, the continued slow growth of the layer leads to capacity fade, and therefore the layer is of great interest. Owejan, Owejan *et al.* (2012 ▶) first used neutron reflectometry to study the formation and structure of the layer in a lithium half-cell, as shown in Fig. 10 ▶. A requirement for neutron reflectometry is a flat and smooth surface, as it probes the average in-plane scattering length density profile. The half-cell of Owejan, Owejan *et al.* (2012 ▶) was configured with Cu as the ‘counter’-electrode to prevent Li reaction with the electrode, ensuring that all the electrochemical charge could be attributed to the decomposition of the electrolyte and SEI layer formation. Subsequently, Yonemura *et al.* (2014 ▶) developed a purpose-built cell for such experiments.

#### 3.5.4. *In operando* charge-carrier distribution measurement   

The macroscopic distribution of charge carriers throughout a battery is of great interest for the optimization of battery design, with neutron imaging methods offering the opportunity to obtain this information. Currently, neutron radiography is limited by spatial and temporal resolution, but developments in instrumentation are likely to improve this for *in operando* research. The spatial variation in discharge products across the bulk of a lithium–air electrode was reported using neutron tomography (Nanda *et al.*, 2012 ▶). Neutron imaging finds a non-uniform lithium distribution across the electrode thickness, with a higher lithium concentration near the edges of the lithium–air electrode and a more uniform concentration at the centre, an observation attributed to polarization factors. Senyshyn *et al.* (2012 ▶) used neutron tomography to examine commercial lithium-ion cells of the 18650 type, an annular design where electrode layers are rolled around a central pin. This work revealed a pronounced contrast in the lithium distribution between the layers in the discharged state, attributed to inhomogeneities resulting from the significant amount of lithium situated inside the positive electrode, where the negative electrode (graphite) is nearly lithium free. During charge, this contrast vanished. Senyshyn *et al.* (2014 ▶) also used spatially resolved *in operando* diffraction in which a gauge volume was defined from which diffraction data were recorded, to probe these lithium distributions. Owejan, Gagliardo *et al.* (2012 ▶) achieved 14 µm spatial resolution in the neutron imaging of batteries using a specialist cell in which the lithium content during charge/discharge and the residual could be measured after each cycle. Changes in the lithium distribution in the battery during cycling and between cycles revealed differences between the lithium concentrations in the separator and current collector.

Another method of determining lithium concentration in materials is neutron depth profiling. Typically, the spatial resolution of neutron depth profiling for well defined homogeneous layers is tens of nanometres, with the main drawback being the limited depth that can be probed. Oudenhoven *et al.* (2012 ▶) showed for the first time that the evolution of the lithium distribution under dynamic conditions can be studied using neutron depth profiling. This was demonstrated *in operando* using a microbattery consisting of a thin-film solid-state stack. The differences between the neutron depth profiling data for the as-prepared electrode in the charged and discharged spectra directly revealed the changing lithium distribution.

## 4. Outlook   

The increasing performance of X-ray and neutron sources and their associated instrumentation has enabled significant improvements in temporal resolution, pushing the frontiers of *in situ* and *in operando* research. This is expected to continue alongside improvements in specialized instrumentation and sample environments. New beamlines dedicated to *in situ* experiments are being developed (Buffet *et al.*, 2012 ▶; Gann *et al.*, 2012 ▶; Radulescu *et al.*, 2012 ▶; Gilles *et al.*, 2014 ▶; Santoro *et al.*, 2014 ▶) featuring, among others, higher flux and fast and large detectors. Integral to the success of this experimentation is the use of simultaneous and complementary measurements. Although not detailed here, examples include combining imaging ellipsometry and GISAXS for studies of polymer morphologies (Körstgens *et al.*, 2010 ▶, 2012 ▶), and simultaneous conductivity and SAXS experiments for investigating polymer electrolyte membranes (Jackson *et al.*, 2013 ▶). Additionally, a number of sample environments have been developed which enable a variety of *in situ* investigations, such as a spray deposition chamber for film formation (Al-Hussein *et al.*, 2013 ▶), stopped-flow systems for the mixing of different components (Grillo, 2009 ▶), solvent vapour and humidity chambers (Smilgies *et al.*, 2009 ▶; Gu *et al.*, 2013 ▶; Jackson *et al.*, 2013 ▶), and flow cells (Moulin *et al.*, 2008 ▶; Manet *et al.*, 2011 ▶; Qazi *et al.*, 2011 ▶). Similarly, advances in computational resources allow a more detailed insight into the underlying structure, dynamics and processes being investigated, where the modelling of these is highly complex in whole *in operando* data sets. These advances also help to address the requirement for the analysis of the large volumes of data resulting from high spatial and temporal resolution.
